# Third-Party Testing Nutritional Supplement Knowledge, Attitudes, and Use Among an NCAA I Collegiate Student-Athlete Population

**DOI:** 10.3389/fspor.2020.00115

**Published:** 2020-09-15

**Authors:** Kaila Ann Vento, Floris Cornelis Wardenaar

**Affiliations:** College of Health Solutions, Arizona State University, Phoenix, AZ, United States

**Keywords:** dietary supplement, anti-doping, third-party testing, education, safety

## Abstract

Dietary supplements, sports foods, and ergogenic supplements are consumed to increase performance, recovery, and health, but risk contamination with illegal substances. Third-party testing programs may assist in regulating the purity and safety of supplements, yet athletes' attitudes and use of such programs are not widely reported. This study examined nutritional supplement knowledge, attitudes, and use, as well as the purchase of third-party tested supplements among university student-athletes (*N* = 138). Knowledge of nutritional supplements yielded a median and (IQR) score of 25% (17 to 42%). Sixteen percent of student-athletes said they were knowledgeable about supplements and their effects, *p* < 0.001. All athletes stated they used a dietary supplement or sports food at least once within the last 12 months, and 77% consumed at least one “claimed to be” ergogenic supplement. Sixty-six percent of student-athletes purchased nutritional supplements not provided by the athletic department. Females athletes were more likely to consume a combination of vitamins and single minerals, a larger variety of sports foods, exotic berries, herbs, maca root powder, ribose, ephedra, colostrum, and hydroxy-methyl-buterate (HMB) than males. Over 90% believed it was essential to know if a supplement was third-party tested. However, only 57% stated the supplements bought were third-party tested. No sex differences were found for nutritional supplement knowledge, attitudes, and use of third-party testing programs. Our results indicate a need to improve student-athletes' attitudes toward and knowledge of nutritional supplements, and the initiation of programs to assist in the choosing and consuming of third-party tested supplements.

## Introduction

Athletes are motivated to use nutritional supplements to enhance performance, recovery, or health, but a lack of regulation of such supplements leads to doping concerns (Thomas et al., 2016; Mathews, 2017). Nutritional supplements are products for oral consumption that add to the nutritional value of the routine diet (Thomas et al., 2016). In sports, nutritional supplements are characterized into three classifications, dietary supplements, sports foods, and ergogenic supplements (Wardenaar et al., 2017).

Dietary supplements are consumed orally, usually in pill or powder form, to augment one's diet and are intended to improve general health through the prevention and treatment of nutrient deficiencies. They can include vitamins, minerals, herbs, or bioactives (e.g., essential fatty acids, carotenoids, nutritional extracts) (Wardenaar et al., 2017).

Sports foods, such as sports drinks and energy bars, have nutritional value (Wardenaar et al., 2017). In the US, dietary supplements and sports foods are differentiated by legal food class, regulations, and packaging labels. Dietary supplements are labeled as “supplement facts” and sports foods as “nutrition facts.” Athletes frequently use sports foods that deliver energy, electrolytes, or building blocks for training adaptation and recovery (e.g., sports drinks, recovery drinks, and energy and protein bars) (Wardenaar et al., 2017). These products often contain carbohydrates and protein and provide a convenient alternative to regular food.

Ergogenic supplements are specially produced with the intent to aid in performance enhancement or recovery (Thein et al., 1995). Ergogenic supplements are continually evolving, and sound evidence is lacking for the effectiveness of most supplements with claimed ergogenic effects. Substantial supporting scientific evidence has been found for caffeine, creatine, dietary nitrate (i.e., beetroot juice), b-alanine, and sodium bicarbonate (Thein et al., 1995; Wardenaar et al., 2017).

Manufacturers often falsely claim nutritional supplements improve performance and health, increasing consumer demand and market production (Thomas et al., 2016; Mathews, 2017). Over-the-counter nutritional supplements do not need to demonstrate clinical efficacy and are exempt from the Federal Drug Administration (FDA) approval (Thomas et al., 2016; Mathews, 2017). To minimize sales of contaminated products, sports certified third-party testing organizations test the purity and quality of dietary supplement and sport foods before distribution to retail. Third-party testing entails independent third-party organizations performing specific tests verifying the safety of nutritional supplements. Examples of third-party testing organizations include the NSF Certified for Sport (National Sanitation Foundation International, Ann Arbor. MI, USA) and Informed Sport (LCG, Teddington, UK), which test every batch of products before market release and attests products do not contain substances banned by sports organizations, supplement contents match printed labels, and unsafe and doping-related contaminants are not detectable [Informed Sport, 2020; National Sanitation Foundation International (NSF International), 2020]. However, purchasing nutritional supplements remains a threat as most supplements do not undergo third-party testing, putting athletes at risk of consuming products contaminated with doping-related substances.

To protect student-athletes from unintended doping (i.e., taking performing-enhancing drugs to gain an unfair competitive advantage), the US-based National Collegiate Athletic Association (NCAA) follows a food-first approach, endorsing food choices above the use of nutritional supplements [Buell et al., 2013; National Collegiate Athletic Association (NCAA), 2013]. Within a Division I university, the NCAA allows athletic departments to provide permissible supplements to facilitate the essential needs of student-athletes while reducing the chance of a positive doping test (Buell et al., 2013). Permissible products are limited to carbohydrate/electrolyte drinks, bars, carbohydrate boosters, protein supplements, and those including omega-3 fatty acids and some vitamins and minerals (Buell et al., 2013). Products that are not permitted (i.e., impermissible) are those not provided or endorsed by an athletic department such as creatine and weight-gainers (Buell et al., 2013). Banned products, including diuretics and anabolic agents, are prohibited from being used by NCAA student-athletes. Student-athletes are responsible for ensuring all products, permissible and impermissible, are safe for consumption. Third-party testing is a market solution to assist in purchasing safe and quality products. Athletes are urged to use third-party testing programs independent of the product manufacturers and their advertising revenues and to consult with a sports dietitian before use (Wardenaar et al., 2017).

Despite the food-first approach and student-athletes being warned about the risk of contaminated nutritional supplements, previous research examining supplement consumption indicated 88–95% of student-athletes used at least one supplement a month (Burns et al., 2004; Froiland et al., 2004; Kristiansen et al., 2005; Buckman et al., 2013). Athletes use nutritional supplements more than non-athletes in the US (Knapik et al., 2015). Likewise, athletes who regularly consume nutritional supplements are more likely to ingest doping products (Sekulic et al., 2016). Percentages of permissible supplements, vitamins/minerals, consumed were 73% (Burns et al., 2004), 66% vitamins and 39% minerals (Froiland et al., 2004), and 56% (Kristiansen et al., 2005). Sports drinks, gels, and bars were consumed by 75, 30, and 64%, athletes, respectively (Kristiansen et al., 2005). Impermissible supplements found by Burns et al. (2004) included herbal supplements (23%) and creatine (31%). Froiland et al. (2004) documented herbal (26%) and creatine (37%) use to be similar. Kristiansen et al. (2005) found creatine use to be lower (5%). Banned supplements, such as caffeine, were reported by 11% (Froiland et al., 2004) and 76% (Kristiansen et al., 2005) of athletes. Impermissible and banned supplement use is higher among male athletes (e.g., amino acids, creatine, dehydroepiandrosterone often abbreviated as DHEA) while females consumed more permissible supplements (e.g., multivitamin, calcium, and iron) (Froiland et al., 2004; Kristiansen et al., 2005; Wardenaar et al., 2016). Females consumed supplements more for health and males for performance and muscle gains (Froiland et al., 2004), yet reasons to consume a particular dietary, sports food, or ergogenic supplement were similar (Wardenaar et al., 2017). Of these studies, no reports were given as to whether the supplements student-athletes used were third-party tested (Froiland et al., 2004; Kristiansen et al., 2005; Wardenaar et al., 2017).

Few studies have examined collegiate student-athletes supplement knowledge (Jessri et al., 2010; Torres-McGehee et al., 2012; Weeden et al., 2014). The supplement knowledge scores reported by Jessri et al. (2010), Torres-McGehee et al. (2012), and Weeden et al. (2014) are 30, 66, and 33%, respectively. (Trakman et al., 2016) examined the quality of these instruments. It was revealed that the instruments administered by Jessri et al. (2010), Torres-McGehee et al. (2012), and Weeden et al. (2014) were either self-developed or modified, lacked documentation of instrument psychometric properties, and supplements questions were too few and did not regard third-party testing (Thomas et al., 2016). The Nutrition for Sport Knowledge Questionnaire (NSKQ) was developed and validated by Trakman et al. (2017) to address these concerns, which included a supplements subscale with two third-party testing questions. She found median supplement knowledge scores for Australian athletes with and without nutrition education of 33 and 25%, respectively (Trakman et al., 2017). Supplement knowledge differences between males and females have not been examined (Jessri et al., 2010; Torres-McGehee et al., 2012; Weeden et al., 2014; Trakman et al., 2017). Athletes' knowledge regarding nutritional supplement risks highlights the importance of a primary education program to help protect collegiate athletes from becoming victims of unintentional doping, illness, and a threat to their student-athlete eligibility status (Knapik et al., 2015).

Preventing the consumption of ill-manufactured products is essential and evidence-based curricula to educate student-athletes about nutritional supplements are needed. Current nutrition education curricula within athletics improve the quality of dietary intake, yet they contain little information about supplements and third-party testing programs (Valliant et al., 2012; Parks et al., 2016; Coccia et al., 2020). The NCAA and NATA consider it necessary to develop nutritional supplement education programs (Buell et al., 2013). While previous research has assessed athletes' knowledge (Jessri et al., 2010; Torres-McGehee et al., 2012; Wiens et al., 2014; Trakman et al., 2017) and usage of supplements (Burns et al., 2004; Froiland et al., 2004; Kristiansen et al., 2005; Knapik et al., 2015), the relationships between current knowledge, attitudes, and use in conjunction with the use of third-party testing are unknown within US collegiate athlete population.

Understanding the relationships of these critical variables may enable effective curricula. Our first aim was to assess collegiate student-athletes' knowledge of, attitudes toward, and use related to nutritional supplements. To the best of our knowledge, student-athlete attitudes toward and usage of third-party testing programs have not been evaluated. Based on previously reported results in collegiate athletes (Burns et al., 2004; Froiland et al., 2004; Kristiansen et al., 2005; Trakman et al., 2017), we hypothesized that student-athlete knowledge of nutritional supplements would be similar to that found in other athletes (score between 25 and 30%) (Trakman et al., 2017), their attitude toward them positive, and their purchasing and consumption high. In which high is defined as 75% of the athletes are consuming at least one or more dietary supplements, sports food or ergogenic supplement over the last 12 months. Further, we hypothesized that athletes' knowledge of third-party testing would be low, their attitude toward third-party testing, therefore indifferent, but we expected that athletes familiar with third-party testing would be likely to purchase their supplements through this system. Sex comparisons were examined, and based on the findings of Wardenaar et al. (2017), they were not expected to differ on supplement knowledge, attitudes, and use substantially.

## Materials and Methods

### Participants

Student-athletes (*N* = 138; *n* = 89 female, and *n* = 49 male) from a public Southwest university participated in this study (athletic department size, N≈ 600). Eligibility included student-athlete status, a minimum age of 18, and participation in a NCAA Division I sport. All completed an informed consent document before receiving a web-based link to the study questionnaires (Qualtrics, 2017). The University's Institutional Review Board (STUDY00008336) and the Research and Technology Committee of the University's athletic department approved the study.

### Procedures

Data collection took place in two phases that differed slightly in the recruitment strategy. In phase one (November 2018), we used face-to-face recruitment during arranged sport strength and condition training sessions. In phase one, the research team was unable to arrange a meeting with each of the sports teams. A recruitment strategy was modified to ensure all student-athletes were aware of the opportunity to participate in a research study. E-mail invitations were sent by athletic personnel in Phase two (February 2019). The modifications in phase two lead to an increase in participation. In both phases, student-athletes were provided a brief description of the study, along with a paper or electronic signed informed consent. After obtaining informed consent, student-athletes completed the anonymous questionnaires (15–20 min) and received a $10.00 electronic gift card.

### Measures

The questionnaire consisted of four categories: general characteristics, knowledge of and attitude toward nutritional supplements, and use.

#### General Characteristics

Participants reported age, sex, sport, and training hours per week during the in- and off-season, along with the presence of dietary counseling and the number of dietary counseling visits.

#### Knowledge of Nutritional Supplements

The supplements subscale (*n* = 12 items) from the NSKQ was used to assesses self-reported supplement knowledge (Trakman et al., 2017). Questions are formatted as multiple-choice and disagree/agree/not sure with a total score range between 0 and 12 (Trakman et al., 2017). The supplements subscale has internal reliability of KR-20 of 0.69 and test-retest reliability of *r* = 0.60 (Trakman et al., 2017). We found a Cronbach's alpha for the supplements subscale to be 0.60, revealing questionable internal consistency (Santos, 1999). Question deletion assessing Cronbach's alpha deemed not to increase the internal consistency of the supplements subscale.

#### Attitudes Toward Nutritional Supplements

Self-perceived importance of diet quality, sports foods, and dietary/ergogenic supplements were rated on an 11-point Likert scale (range 0–10). Self-perceived knowledge of sports foods, dietary/ergogenic supplements, and NCAA banned supplements, and their effects were asked through yes/no responses. Interest in learning more about nutritional supplements and types were requested. From a predetermined list, student-athletes selected the top five nutritional sources, such as a dietitian, coach, and teammate, influencing diet and supplement use.

#### Nutritional Supplement Use

We assessed the use of and reason (i.e., improve health, performance, or both) for consuming 50 pre-specified nutritional supplements (i.e., vitamins/minerals, sports foods, and ergogenic aids) over the last 12 months based on a previous questionnaire of Wardenaar et al. (2016).

The responses of two questions from the NSKQ supplements subscale (e.g., “The purity and safety of all supplements are tested before sale” and “Supplement labels may contain false or misleading information”) were examined in more detail. Student-athletes were asked if they consumed nutritional supplements not provided by the athletic department, and if so, who purchased them (i.e., themselves, parents, together with parents, and other), and whether those supplements were third-party tested. The proportion of student-athletes consuming third-party tested supplements and answering the above questions correctly were further examined, preferably with a 75% outcome.

### Statistical Analysis

Descriptive data for general characteristics, nutritional supplement attitudes and use, and third-party testing are reported as frequencies (*n*), percentages (%), and mean ±*SD*. The self-reported supplement knowledge scores are given as the median and interquartile range (IQR) due to non-normally distributed data. A Mann–Whitney *U*-test examined differences in supplement knowledge scores between male and female athletes. Chi-square tests of independence examined proportional sample and sex differences concerning nutritional supplement attitudes and use and third-party testing. The statistical significance level was set at *p* ≤ 0.05. Data were analyzed utilizing IBM SPSS 25 (version XVII; SPSS Inc, Chicago, IL).

## Results

### General Characteristics

The top three sports represented were swimming/diving (30%, *n* = 41), track and field (15%, *n* = 21), and soccer (12%, *n* = 16). Student-athletes (20 ± 1.6 years of age) trained 20.6 ± 6.5 hours per week in-season and 15.5 ± 7.2 h off-season. Self-reported training hours did not significantly differ between males and females, *p* > 0.05. See Table 1 for additional group and sex characteristics.

**Table 1:** General characteristics of the responding student-athletes.

	**Total**	**Female**	**Male**
% (*N*)	100 (138)	65 (89)	35 (49)
Age	19.8 ± 1.6^a^	19.7 ± 1.4^a^	20.1 ± 1.8^a^
Training hours			
In-season	20.6 ± 6.5^a^	20.6 ± 5.7^a^	20.6 ± 7.7^a^
Off-season	15.5 ± 7.2^a^	15.3 ± 7.5^a^	15.9 ± 6.8^a^
Sport % (*n*)
Swimming/Diving	30 (41), *r* = 68^b^	18 (16), *r* = 30^b^	51 (25), *r* = 38^b^
Track and field	15 (21), *r* = 76^b^	16 (14), *r* = 36^b^	14 (7), *r* = 40^b^
Soccer	12 (16), *r* = 32^b^	18 (16), *r* = 32^b^	
Lacrosse	10 (14), *r* = 38^b^	16 (14), *r* = 38^b^	
Beach volleyball	6 (8), *r* = 20^b^	9 (8), *r* = 20^b^	
Basketball	4 (5), *r* = 31^b^	5 (4), *r* = 14^b^	2 (1), *r* = 14^b^
Triathlon	4 (5), *r* = 11^b^	5 (5), *r* = 11^b^	
Softball	2 (3), *r* = 18^b^	3 (3), *r* = 18^b^	
Tennis	2 (3), *r* = 10^b^	3 (3), *r* = 10^b^	
Cross-country	1 (2), *r* = 25^b^	1 (1), *r* = 11^b^	2 (1), *r* = 17^b^
Ice hockey	5 (7), *r* = 28^b^		14 (7), *r* = 28^b^
Wrestling	4 (6), *r* = 25^b^	1 (1), *r* = 11^b^	12 (5), *r* = 17^b^
Football	2 (3), *r* = 88^b^		5 (3), *r* = 88^b^
Missing^*^	3 (4)	5 (4)	

a *Mean ± SD*.

b *r = roster size*.

* *Four participants did not fill out question. All percentages rounded to nearest whole number*.

Fifty-eight percent (*n* = 80) reported 1–2 team or individual meetings with a registered sports dietitian during the last 12 months, and 13% reported three or more contacts. Sixty-eight percent (*n* = 94) visited with a registered sports dietitian within the athletic department, and 11% (*n* = 10) visited a registered dietitian not affiliated with the athletics department. No relationship was found between purchasing third-party tested supplements and the number of visits with a registered dietitian with or without affiliation with the athletic department, *p* > 0.05.

### Knowledge of Nutritional Supplements

The median and IQR for the self-reported supplement knowledge score was 25% (17 to 42%). No significant differences between males and females' supplement knowledge scores were found, *p* = 0.42. The median and IQR for the self-reported supplement knowledge score for males and females was 25% (17 to 42%) and 25% (17 to 33%), respectively.

### Attitudes Toward Nutritional Supplements

The self-reported importance of overall diet quality, sports foods, and dietary/ergogenic supplements to their diets was 8.1 ± 1.9, 5.9 ± 2.4, and 4.5 ± 2.7, respectively (range; 0–10). No differences were found between male and female athletes' perceived importance of overall diet quality, sports foods, and dietary/ergogenic supplements to their diets, *p* > 0.05. Forty-nine percent believed they were knowledgeable about sports foods and their effects. Few (16%, *n* = 2) stated to be knowledgeable about dietary/ergogenic supplements with performance enhancements claims and their effects, *p* < 0.001. Thirty percent (*n* = 41) stated to be knowledgeable about NCAA banned supplements and their effects, *p* < 0.001. No differences were found between male and female athletes' perceived knowledge of sports foods, dietary/ergogenic supplements, and NCAA banned supplements and their effects, *p* > 0.05. Seventy percent (*n* = 96) expressed no interest in learning more about specific supplements, although 93% (*n* = 129) stated it was important for athletes to know if supplements are tested for banned substances. The top listed supplements by those interested in learning more (*n* = 41) were creatine (34%), caffeine (10%), Branched Chain Amino Acids (BCAA's) (7%), and collagen (5%). The top five influences on dietary choice and supplement use were the dietitian/nutritionist (92%), athletic trainer (64%), personal trainer (48%), physician (45%), and coach (43%).

### Nutritional Supplements Use

All (*N* = 138) completed the dietary supplement use section. Thirteen participants did not properly complete the sports foods and ergogenic supplements use sections, and the responses of these athletes were eliminated from the sports foods and ergogenic supplement sections only due to incomplete data. Table 2 lists the reported supplements and reasons for consumption, while also noting NCAA classification (i.e., permissible, impermissible, and banned) for the nutritional supplements included.

**Table 2:** Reported usage and reasons to take nutritional supplements within the last 12 months.

**Supplement**	**Reported using % (*n*)**	**Improve health %**	**Improve performance %**	**Both %**
Dietary supplements (*N* = 138)				
Multivitamin & mineral supplement	65 (89)	38	7	55
Single vitamins	64 (88)	42	5	53
Single minerals	63 (87)	26	10	64
Combination of vitamins	52 (72)	29	14	57
Fish oil or essential fatty acids	52 (72)	43	7	50
Combination of 1–3 vitamins & minerals	46 (63)	27	14	59
Combination of minerals	43 (60)	23	15	62
Sport foods (*N* = 125)				
Sports drinks	94 (117)	4	58	38
Recovery drinks	90 (112)	12	34	54
Energy bar	89 (111)	10	46	44
Chocolate milk	77 (96)	17	34	49
Protein shake	66 (83)	6	41	53
Energy gel	65 (82)	2	72	24
Protein bar	60 (75)	9	36	55
Energy drink	54 (67)	*9*	69	22
Maltodextrin	32 (40)	7	45	48
Weight gainer	31 (39)	15	39	46
Ergogenic supplements (*N* = 125)				
*Caffeine^a^*	*63 (79)*	*8*	*58*	*34*
Tart cherry (juice) or as supplement	39 (49)	8	39	53
Probiotics rich foods or as supplement	32 (40)	28	5	67
Fresh exotic berries or as supplement	29 (36)	25	17	58
Creatine	29 (36)	3	55	42
BCAA	22 (28)	6	31	63
Dietary nitrate rich foods or as supplement	21 (27)	7	32	61
Herbs	20 (25)	26	22	52
Beta-alanine	19 (24)	8	40	52
Leucine	19 (24)	4	42	54
Maca root powder	17 (21)	17	8	75
Sodium bicarbonate	17 (21)	0	43	57
CLA	16 (20)	10	33	57
MCT	15 (19)	20	20	60
Glucosamine/ Chondroitine	15 (19)	16	31	53
L-carnitine	15 (19)	16	31	53
Curcumin	14 (18)	26	26	48
Ribose	14 (18)	11	39	50
Quercetin	*13 (17)*	5	39	56
*Ephedra*	13 (17)	*12*	*47*	*41*
Tribulus Terrestris	*13 (17)*	6	47	47
*DHEA*	13 (17)	*12*	*35*	*53*
Glycerol	*12 (16)*	18	29	53
*Colostrum*	12 (16)	*6*	*44*	*50*
HMB	12 (16)	12	44	44

*Underlined NCAA impermissible products. Italicized NCAA banned products. All others are classified as NCAA permissible products based on the following classification: carbohydrate/electrolyte drinks, bars, carbohydrate boosters, protein supplements, and those including omega-3 fatty acids and some vitamins and minerals. All percentages rounded to the nearest whole number*.

a *Caffeine permissible to a urine metabolite level of 15 ug/mL*.

All student-athletes (*N* = 138) reported consuming at least one dietary supplement within the last 12 months. The most-reported were multivitamin and mineral supplements (65%) and single vitamins or minerals (64 and 63%, respectively). Improving health and performance were the main reasons for using these dietary supplements (53 and 64%, respectively). Female athletes were more likely to consume a combination of vitamins (*p* < 0.01) and single minerals (*p* < 0.05) compared to males (see Table 3).

**Table 3:** Reported usage of nutritional supplements between male and female athletes.

	**Female (*n* = 89)^b^**	**Male (*n* = 49)**
**Supplement**	**Reported using % (*n*)**	**Reported using % (*n*)**
Dietary supplements (*N* = 138)		
Multivitamin & mineral supplement	66 (59)	63 (31)
Single vitamins	65 (58)	61 (30)
Single minerals^*^	70 (62)	51 (25)
Combination of vitamins^**^	61 (54)	37 (18)
Fish oil or essential fatty acids	46 (41)	63 (31)
Combination of 1-3 vitamins & minerals	52 (46)	35 (17)
Combination of minerals	49 (44)	33 (16)
Sport foods (*N* = 125)
Sports drinks	95 (72)	92 (45)
Recovery drinks	91 (69)	88 (43)
Energy bar^*^	93 (71)	82 (40)
Chocolate milk^*^	83 (63)	67 (33)
Protein shake	70 (53)	61 (30)
Energy gel^*^	74 (56)	53 (26)
Protein bar^*^	67 (51)	49 (24)
Energy drink	61 (46)	43 (21)
Maltodextrin^*^	40 (30)	18 (9)
Weight gainer	37 (28)	22 (11)
Ergogenic supplements (*N* = 125)		
*Caffeine^a^*	*67 (51)*	*57 (28)*
Tart cherry (juice) or as supplement	40 (30)	39 (18)
Probiotics rich foods or as supplement	38 (29)	22 (11)
Fresh exotic berries or as supplement^**^	41 (31)	14 (9)
Creatine	24 (18)	37 (18)
BCAA	25 (19)	33 (16)
Dietary nitrate rich foods or as supplement	25 (19)	18 (9)
Herbs^**^	30 (23)	8 (4)
Beta-alanine	18 (14)	22 (11)
Leucine	20 (15)	18 (9)
Maca root powder^*^	25 (19)	10 (5)
Sodium bicarbonate	20 (15)	5 (6)
CLA	21 (16)	10 (5)
MCT	21 (16)	8 (4)
Glucosamine/Chondroitine	20 (15)	8 (4)
L-carnitine	20 (15)	8 (4)
Curcumin	20 (15)	8 (4)
Ribose^*^	20 (15)	6 (3)
Quercetin	18 (14)	8 (4)
Ephedra^*^	*20 (15)*	*4 (2)*
Tribulus Terrestris	18 (14)	6 (3)
*DHEA*	*18 (14)*	*6 (3)*
Glycerol	18 (14)	6 (3)
Colostrum^*^	*18 (14)*	*4 (2)*
HMB^*^	18 (14)	4 (2)

*Underlined NCAA impermissible products. Italicized NCAA banned products. All others are classified as NCAA permissible products based on the following classification: carbohydrate/electrolyte drinks, bars, carbohydrate boosters, protein supplements, and those including omega-3 fatty acids and some vitamins and minerals. All percentages rounded to the nearest whole number*.

a *Caffeine permissible to a urine metabolite level of 15 ug/mL*.

b *Missing n = 13 female athlete sports foods and ergogenic supplement use*.

* *P < 0.05*.

** *P < 0.01*.

One hundred percent (*n* = 125) reported consuming at least one sports food product within the last 12 months, and they most frequently used sports drinks (94%), recovery drinks (90%), and energy bars (89%) These sports food products are permitted by the NCAA and provided by the athletic department. Respondents mostly consumed recovery drinks, energy bars, and sports drinks to improve performance, 34%, 46%, and 58%, respectively. More female athletes reported energy bars, chocolate milk, energy gels, protein bars, and Maltodextrin *p* < 0.05 (see Table 3).

Ergogenic supplement use was reported by 77% (*n* = 125), and the top three products consumed were caffeine (63%), tart cherry (39%), and probiotics (32%). Fifty-eight percent used caffeine to improve performance, and they used tart cherry and probiotics to improve both performance and health, 53–68%, respectively. Tart cherry was provided as a juice by the athletic department. Supplements not permitted by the NCAA (e.g., Tribulus Terrestris) reported banned (e.g., DHEA, ephedra, and colostrum) were used by relatively few athletes (12–14%). Table 2 displays a complete list of reported uses and reasons for using dietary supplements, sports foods, and ergogenic supplements. The female athletes reported more frequently the use of fresh exotic berries and herbs (*p* < 0.01) as well as maca root powder, ribose, ephedra, and colostrum (*p* < 0.05) (see Table 3).

**Figure 1:**
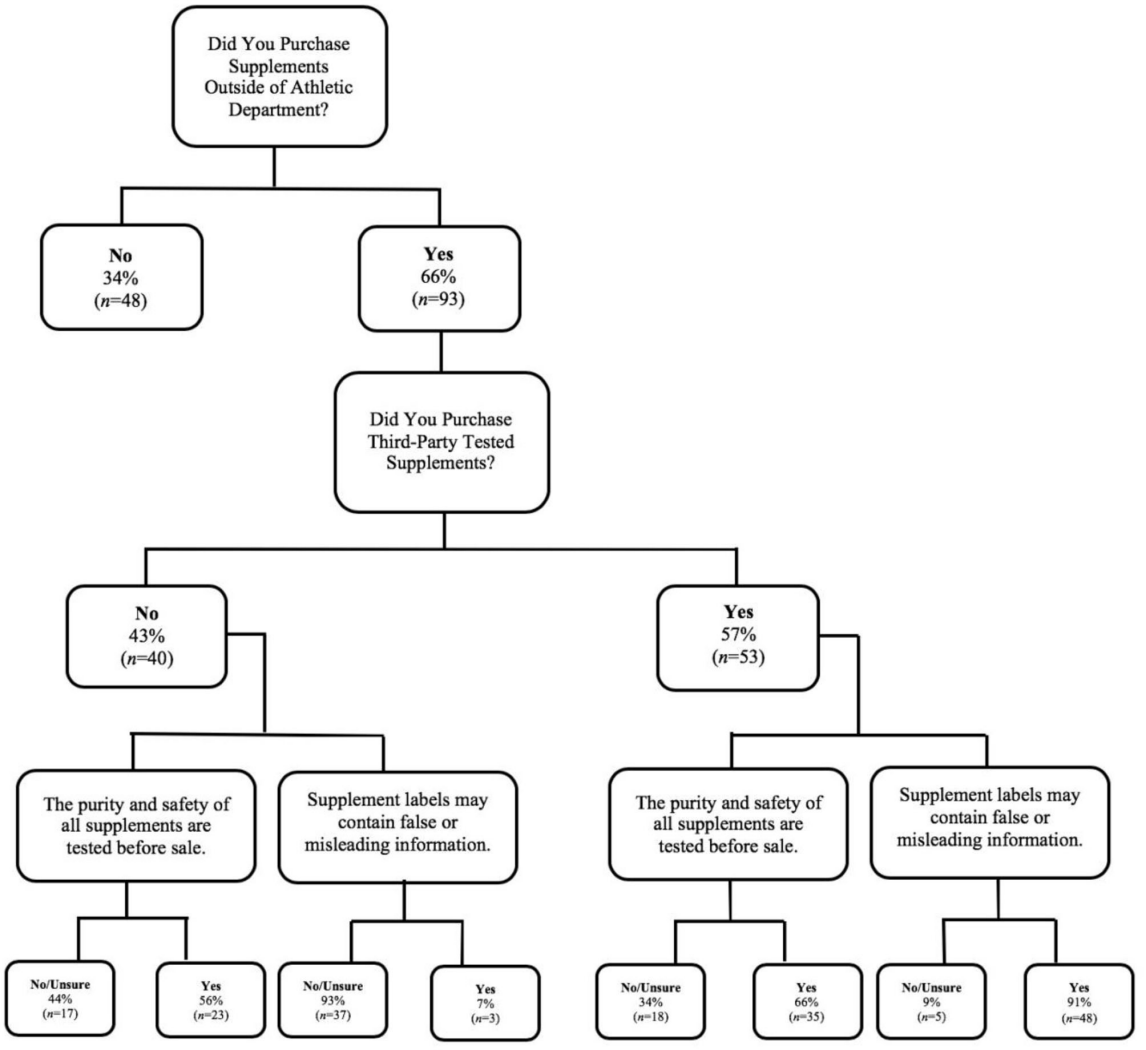
Student-athletes' purchasing and knowledge of third-party tested supplements.

The following percentages are regarding specific knowledge questions in the NSKQ supplement subscale related to third-party testing and purchasing or acquired behaviors. For the breakdown of these percentages, see Figure 1. When asked, “The purity and safety of all supplements are tested before sale,” 62% agreed not all nutritional supplements were, in fact, so tested, *p* < 0.01. Likewise, 90% agreed with the statement, “Supplement labels may contain false or misleading information,” *p* < 0.001. No differences were found between males and females' answers, *p* > 0.05. Sixty-six percent purchased nutritional supplements not provided by the athletic department. These were purchased by themselves (59%), parents (24%), together with parents (16%), or roommate (1%). Fifty-seven percent (males, *n* = 27; females, *n* = 26) stated the supplements they bought were third-party tested. Percentages of male and female athletes consuming supplements not provided by the athletic department and third-party tested were not significantly different, *p* > 0.05. Of those purchasing third-party tested supplements, 43% did not think that all nutritional supplements were tested before sale and that labels contained accurate information; no differences were seen between males and females, *p* > 0.05.

## Discussion

Males and females both reported a median 25% supplement knowledge score, comparable to the previous reporting of (Trakman et al., 2017). We found all student-athletes consuming at least one nutritional supplement over the last 12 months. Additionally, over 75% of the athletes consumed one or more ergogenic supplements. Athletes were motivated to consume nutritional supplements for health and performance-enhancing benefits, as reported earlier (Froiland et al., 2004; Kristiansen et al., 2005). Student-athletes are aware of the importance of using third-party tested supplements. However, just over half (males and females equally) reported buying these third-party tested supplements when they purchased nutritional supplements.

Nutritional supplement consumption of multivitamins (65%), sports drinks (94%), caffeine (63%), and herbs (21%) was similar to the findings of Burns et al. (2004), Froiland et al. (2004), Kristiansen et al. (2005). Permissible, impermissible, and banned supplement use was higher in female than male athletes, which may be the result of unequal group sizes between males and females (*p* < 0.001). It has been shown that sex differences in supplement use between high-level athletes are limited to, for example, prescribed iron micronutrients (Wardenaar et al., 2017). The differences in reported use for sports foods may be the result of preference differences between males and females, as both sexes reported the use of functional foods helping them to fuel their performance (sports drinks containing carbohydrate) and recovery (protein-containing products).

The NCAA does not endorse any nutritional supplements, and despite regulating supplements as permissible, impermissible, and banned, intercollegiate athletes report many products outside the permissible category (Burns et al., 2004; Froiland et al., 2004; Kristiansen et al., 2005; Wardenaar et al., 2017). Amongst the most frequently used impermissible supplements were caffeine and BCAA's. Caffeine is likely an effective ergogenic, but there is a risk of testing positive when high amounts of caffeine metabolite are present in urine (De Hon and Coumans, 2007). Likewise, BCAA's can be associated with products high in contaminates due to a lack of regulation (De Hon and Coumans, 2007).

Three-fourths of the student-athletes reported occasional (1–2) contact visits during the year with their registered sports dietitian. Wardenaar et al. (2017) found that consistent monthly consultations concerning supplements lead to elite and sub-elite Dutch athletes making more evidence-based, fit for individual use, nutritional supplement choices. Yet, based on the number of student-athletes using nutritional supplements that are not third-party tested, discussions regarding supplements may be limited within this cohort of athletes. In our opinion, at least two factors potentially contributing to the high unregulated use of impermissible supplements obtained from non-third party tested vendors within collegiate athletics. First, most US athletic departments with a sports dietitian on staff follow a food-first approach (Buell et al., 2013). Improving diet quality through foods may take precedence over the use of nutritional supplements. Consultations with the sports dietitian may likely focus on broad nutritional topics, reducing a discussion of the use of supplements to a minimum (Thomas et al., 2016). Secondly, NCAA intercollegiate athletic departments are not allowed to endorse impermissible supplements, yet a high number of intercollegiate athletes reported the use of these supplements (Burns et al., 2004; Kristiansen et al., 2005; Knapik et al., 2015). The NCAA not clearly defining “endorsement of supplements” that may lead to mixed interpretations within and between athletic departments. It is plausible that infrequent consultations and, in our opinion, the current NCAA approach of permissible vs. impermissible nutritional supplements, may impair discussions between student-athletes and sport dietitians. Student-athletes may use an “impermissible” product on their own; however, sports dietitians may feel conflicted on how to advise responsible selection and use of an impermissible supplement at the individual athlete level. Therefore, future studies should consider examining registered sports dietitians' perceptions of how the NCAA's “non-endorsement” position impacts nutritional supplement discussions and means to improve the quality of their work to enable reductions in supplement use.

The solution lies with cultivating an environment in which student-athletes and athletic departments feel safe to address nutritional supplements. Differentiating the terms “endorsement” vs. “discussion” of impermissible supplements might well-increase registered dietitians' willingness to engage in meaningful dialogue while consulting with student-athletes. Athletic departmental efforts in developing an education program available to all student-athletes and staff may initiate an open discussion and reduce hiding the of consumption of not evidence-based non-third-party tested nutritional supplements (Parks et al., 2016). Educational topics should clarify supplement regulations and the importance of third-party testing, and they should be presented in a manner that increases student-athlete and athletic staff interaction. Role-playing, small group activities, and case studies foster engagement and comfort, potentially contributing to positive future discussions of supplement use (McKeachie and Svinicki, 2013). These suggestions may prevent NCAA student-athletes from all divisions (I, II, and III) from consuming unregulated supplements, support dietitians' goals and needs, and increase the sports dietitian and all athletic professionals' knowledge of nutritional supplements (Torres-McGehee et al., 2012).

Sports organizations at the high school, amateur, and professional levels implementing drug-free sports may also benefit from a nutritional supplement education program. The World Anti-Doping Agency (WADA) and the U.S. Anti-Doping Agency's (USADA) seek a doping-free sports environment and formulate and enforce drug policies within sports organizations. WADA and USADA also provide excellent tools and evidence-based research for athletes of all levels to reference. Resources include up-to-date lists of prohibited supplements, medication screenings, descriptions of urine and blood testing, and codes of conduct. Athlete education, prevention, and outreach programs are readily available so that sports organizations can adhere to their specific regulations [U.S. Anti-Doping Agency (USADA), 2020; World Anti-Doping Agency (WADA), 2020].

The results of this study support the need for continued efforts in improving student-athlete nutritional supplement knowledge, as knowledge has been relatively low for the last decade (Jessri et al., 2010; Torres-McGehee et al., 2012; Weeden et al., 2014; Trakman et al., 2017). Our study provides a greater understanding of student-athletes use of third-party testing programs. While third-party testing was perceived positively by student-athletes, athletes did not always consider this option when purchasing. Future nutritional supplement educational programs and monitoring systems may improve discussions about permissible vs. impermissible supplements and allocating third-party tested supplements.

The study was not without its limitations, as self-reported data can be biased. Several dietary supplements (i.e., single vitamins) were clustered, and participation from certain sports was lacking. Based on the findings from Wardenaar et al. (2017), athletes reported micronutrient supplements are not always compliant with daily use. As such, the reported frequency cannot be directly converted to specific quantities consumed, making contamination and doping challenging to assess. Also, the “other” option was seldom and limited student-athletes specificity in answers. The open-ended question, to list supplements they were interested in learning more about, was not answered by all, perhaps due to not wanting to invest additional time completing the questionnaire. Generalizability was affected, as a minimal number of male student-athletes from traditional American sports (i.e., basketball, football, and baseball, anecdotally notorious for supplement use participated in the study due to lack of interest. The lower participation from male sports negatively affected group sizes and comparison between male and female athletes.

In conclusion, a low level of knowledge toward third-party testing programs could lead to poor purchases and use. Results from this study may contribute to future studies discovering the barriers in purchasing third-party tested supplements. Initiatives to reduce the risk of student-athletes using unsafe or non-third party tested program could entail a departmental nutritional supplement program to educate, discuss, and monitor use. Increasing knowledge about and attitudes toward consumption of quality nutritional supplements go beyond a student's athletic career. They are important in making safe and healthy nutritional supplement choices throughout one's life.

## Data Availability Statement

All datasets generated for this study are included in the article/Supplementary Material.

## Ethics Statement

The studies involving human participants were reviewed and approved by Arizona State University Institutional Review Board. The patients/participants provided their written informed consent to participate in this study.

## Author Contributions

FW designed the study, data were collected, and analyzed by KV and FW. KV and FW undertook data interpretation and manuscript preparation. All authors approved the final version of the paper.

## Conflict of Interest

The authors declare that the research was conducted in the absence of any commercial or financial relationships that could be construed as a potential conflict of interest.
